# Steel slags for enhanced removal of landfill leachate in a three-dimensional electrochemical oxidation system

**DOI:** 10.1038/s41598-023-39638-w

**Published:** 2023-08-07

**Authors:** Lichao Nengzi, Rui Cao, Yong Qiu, Lin Meng, Wujia Hailai, Haitao Li, Guanglei Qiu

**Affiliations:** 1 Academy of Environmental and Economics Sciences, Xichang University, Xichang, 615013 China; 2 School of Environment and Energy, South China University of Technology, Guangzhou, 510006 China; 3 College of Resources and Environment, Xichang University, Xichang, 615013 China

**Keywords:** Environmental sciences, Chemistry

## Abstract

In this study, a three-dimensional electrochemical oxidation system, with steel slags as particle electrodes, was applied to deal with landfill leachate. The characteristics of particle electrodes were investigated by scanning electron microscope (SEM), X-ray fluorescence spectroscopy (XRF) and X-ray diffraction (XRD) measurements. It was found that the steel slag exhibited rough and irregular surface and mainly consisted of SiO_2_ (Quartz), which indicated the enhanced absorbed and electroconducted abilities. Subsequently, comparative degradation tests between two-dimensional (2D) and three-dimensional (3D) electrochemical oxidation systems were carried out and results indicated removal efficiencies of COD. Moreover, NH_4_^+^-N from landfill leachate in 3D system was greatly improved compared with that of 2D system. Besides, operating conditions were also optimized to interelectrode distance of 1 cm, current density of 20 mA·cm^−2^, initial pH value of 4.4 and steel slag concentration of 0.30 g·mL^−1^, all of which were determined to guarantee excellent landfill leachate removal efficiency. In addition, a possible removal mechanism for this system was proposed. The introduction of steel slag particle electrodes in three-dimensional electrochemical oxidation system implied the concept for “using waste to treat waste”, providing a workable way in pollutant elimination.

## Introduction

Global municipal solid waste (MSW) generation is increasing along with population growth, increased living standards as well as industrialization, attaining a value of 2.2 billion tons per year by 2050^1^. The burial of MSW in Landfills cause high quantity of landfill leachate (LFL), generated when excess precipitation infiltrates through many layers of the landfill^2^. Moreover, LFL contains high levels of organic contaminants, inorganic salts, heavy metals and ammonia^3^, which can cause significant damage to the entire ecological system and human health^4^. Thus, in order to prevent contamination of water resources, surface and groundwater, and soils, adequate collection and treatment of these effluents are needed.

Most of conventional biological and physicochemical technologies implemented at the MSW management facilities at present are not capable to efficiently treat LFL, owing to the inhibitory effect of microbial system caused by certain toxic substances, as well as expensive costs of investment and maintenance^5,6^. Advanced oxidation processes (AOPs) that can produce active species to oxidize refractory organic pollutants have attracted much attention, due to their potential applications towards wastewater removal^7–12^. Among these methods, electrochemical methods have emerged as promising alternatives for wastewater removal especially for LFL^13–16^. During the last two decades, electrochemical oxidation (EO) has made great progress in wastewater treatment, especially for the bio-refractory substance abatement^17^. Generally, EO exhibits many advantages such as lack of sludge production, breakdown of higher molecular compounds to generate biodegradable intermediates and complete mineralization of organics^18^. Moreover, this process has been observed to be efficient for decomposing ammonium, which is considered as the most inflexible pollutant(hard to remove) present in LFL^19^.

Among the EO technologies, three-dimensional electrochemical oxidation (3DEO), with introduced particle electrodes leads to higher specific surface area and shorter distance for mass transfer, which addresses the drawbacks such as low current efficiency and mass transfer limitation encountered by conventional two-dimensional (2D) electrolysis^20–22^. Considering that, selecting proper particle electrodes is a critical factor for designing and operating the 3D electrochemical oxidation system. According to Wang’s study^23^, particle electrodes are mainly prepared as carbonaceous material and metallic (including metal oxide) material with high porosity and high impedance. However, their development and application are limited as they have complicated process and expensive raw materials.

Nowadays, steel slag, which contains several metal oxides and nonmetal oxides (such as SiO_2_, CaO, Fe_2_O_3_ and Al_2_O_3_), has drawn much research attention^24,25^. Steel slag has been widely used as an industrial waste in removing pollutants, owing to its excellent adsorption and sedimentation ability. For example, Cheng et al. used salicylic acid–methanol modified steel slag as Fenton-like catalyst for degradation of alachlor, while^26^ Wang et al.^27^ prepared magnetic steel-slag particle electrodes for 3D electrochemical degradation of oilfield wastewater. However, there are few studies on direct use of steel slag without modification for environmental remediation, especially for LFL degradation.

A three-dimensional (3D) electrochemical oxidation system with steel slags introduced as particle electrodes was thus utilized in the present study for electrochemical treatment of landfill leachate (LFL) from the landfill site located in northwestern China. Physicochemical properties of steel slags were investigated by scanning electron microscope (SEM), X-ray fluorescence spectroscopy (XRF) and X-ray diffraction (XRD). Comparison experiments for the two-dimensional (2D) and three-dimensional (3D) electrochemical oxidation systems were therefore conducted to explore the effect of particle electrodes. Apart from these degradation tests, our experimental work also focused on optimization of degradation process. Various operating parameters, including interelectrode distance, current density, initial pH value and steel slag concentration were optimized to obtain appropriate operating conditions for the system. As expected, the COD and NH_4_^+^-N of LFL were almost completely (over 90%) removed in 120 min under the optimized conditions in the system, with steel slag particle electrodes. This can be named as concept on “using waste to treat waste”. Moreover, the possible removal mechanism was also introduced as well.

## Methods

### Materials

In this work, the steel slags with average size of around 1 cm and volume of steel 4–6 cm^3^, were from obtained iron and steel plant. They were then used as particle electrodes in the 3D electrochemical oxidation system. Sulfuric acid (H_2_SO_4_), potassium dichromate (K_2_Cr_2_O_7_) and ferrous sulfate (FeSO_4_·7H_2_O) were employed for the chemical oxygen demand (COD) measurements. Mercury dichloride (HgCl_2_), Potassium iodide (KI) and potassium hydroxide (KOH) were used for measuring levels of ammonium nitrogen (NH_4_^+^-N). Besides, sodium sulfate (Na_2_SO_4_) and absolute ethanol (C_2_H_5_OH) were used in the experiments. All the chemicals used in this study were of analytical grade and were purchased from Tianjin Kermel Chemical Reagent Co., Ltd (Tianjin, China).

### Description of reaction system

**Figure 1:**
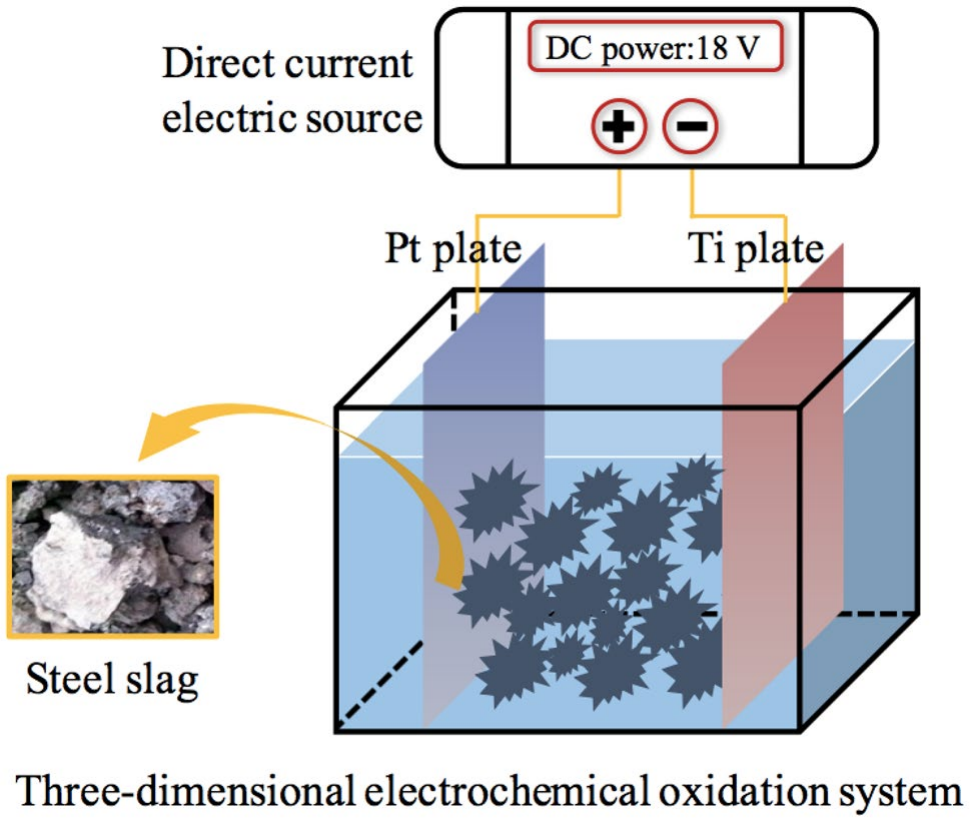
Schematic diagram of three-dimensional electrochemical oxidation system.

The degradation experiment was performed in the three-dimensional (3D) electrochemical oxidation system shown in Fig. 1. As seen, a two-cell configuration was established in the electrolytic tank (70 mm × 45 mm × 45 mm), with Pt and Ti plates (0.5 mm thick) served as anode and cathode, respectively. Besides, the electrodes were positioned vertically and parallel to each other and close to the inside of electrolytic tank. Certain concentration of steel slag particles was filled into the reactor and packed between the two plates in the bottom of the middle chamber to establish a 3D system. The optimized steel slag concentration of 0.30 g·mL^−1^. In addition, a direct current electric source (HY1711-3S, Yaguang) was wired between the anode and cathode, which applied a voltage of 18 V. Moreover, the experiments were conducted in the room temperature of 25 ℃.

For comparison, the two-dimensional (2D) electrochemical oxidation system was performed under the same configurations without packing the particle electrodes.

### Degradation experiment

The experimental landfill leachate served as the degradation project, and was collected from the landfill site without any treatment and stored at 4 °C. Physical and chemical properties of the raw landfill leachate are listed in Table 1, indicating high toxicity and complicated components. The NH_4_^+^-N and COD concentrations of landfill leachate were measured according to Standard Methods for examination of Water and Wastewater (APHA, 2005).

**Table 1:** Characteristics of landfill leachate.

Parameters	CODCr (mg·L^−1^)	NH_4_^+^-N (mg·L^−1^)	pH value
Values ± S.D	13,700 ± 10.3	2250 ± 4.8	8.4 ± 0.3

In this typical experiment, 100 mL of landfill leachate was poured into the micro-electrolysis cell of 3D and 2D electrochemical oxidation system and mixed up with Na_2_SO_4_ solution (10 mL, 0.1 mol·L^−1^) which was employed as supporting electrolyte. The initial pH of solution was adjusted by H_2_SO_4_ (0.1 mol·L^−1^) and NaOH (0.1 mol·L^−1^) and then turned on the DC power to start reaction. During the degradation, approximate 3 mL of the mixture was withdrawn at predetermined intervals and immediately filtered through 0.22 μm membrane for further measurements. Besides, based on the pre-experiments, several influential parameters were considered to optimize the operating conditions of the 3D electrochemical oxidation system, such as interelectrode distance, current density, initial pH value and steel slags concentration.

### Analytical methods

For SEM(JSM-6701F, Hitachi, Japan) test, 10-8 Pa order pressure in Gun, resolution: 1.0 nm(15 kV)/2.2 nm(1 kV), acceleration voltage: 0.5 kV-30 kV, magnification: 25-650 K. Besides, percentages of chemical composition contained in the particle electrodes, which were observed by XRF. The XRD was conducted using a Rigaku D/MAX III-3B diffractometer, with Cu Kɑ irradiation. In addition, COD and levels of NH_4_^+^-N for the landfill leachate were measured according to APHA standard methods, using an ultraviolet-vis (UV–vis) spectrophotometer (EVOLUTION300, Thermofisher Co. Ltd., America). Moreover, the pH values were determined using a pH electrode (pHG-7685A; INESA Instrument Co. Ltd., Shanghai, China).

## Results and discussion

### Characteristics of steel slag particle electrodes

**Figure 2:**
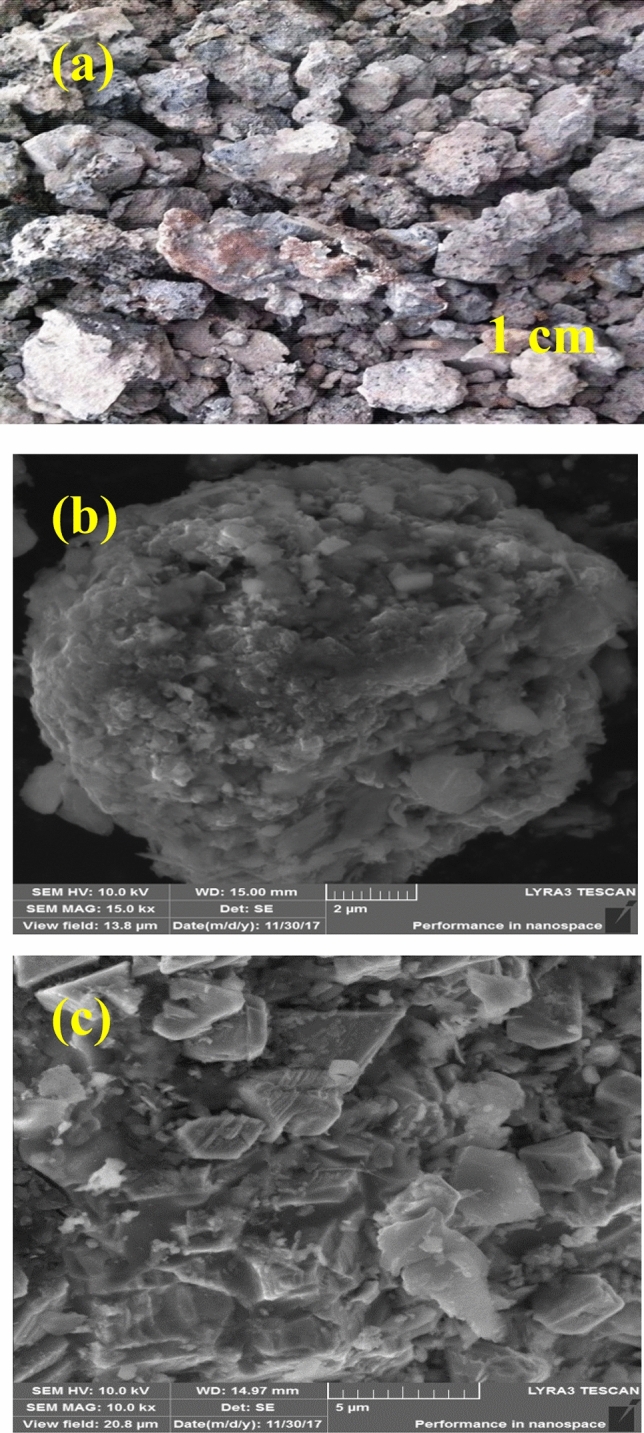
The optical (**a**) and SEM (**b**, **c**) images of steel slag particle electrodes.

The optical and SEM images were employed to study the morphologies of steel slag particle electrodes as displayed in Fig. 2. It was seen from Fig. 2a that there were several rough blocks and particles with different shapes and sizes, coming from steelmaking process and directly used in the three-dimensional electrochemical oxidation system. Besides, the steel slag with average size of around 1 cm was chosen in the typical study. As seen from Fig. 2b, c, the steel slag exhibited micro-scale structure and the sample surface revealed rough and irregular structures. Clearly, there were numbers of sheets and belts inhomogeneously exposed on the surface, which therefore increased the external surface area of steel slags, leading to enhanced adsorption ability.

**Figure 3:**
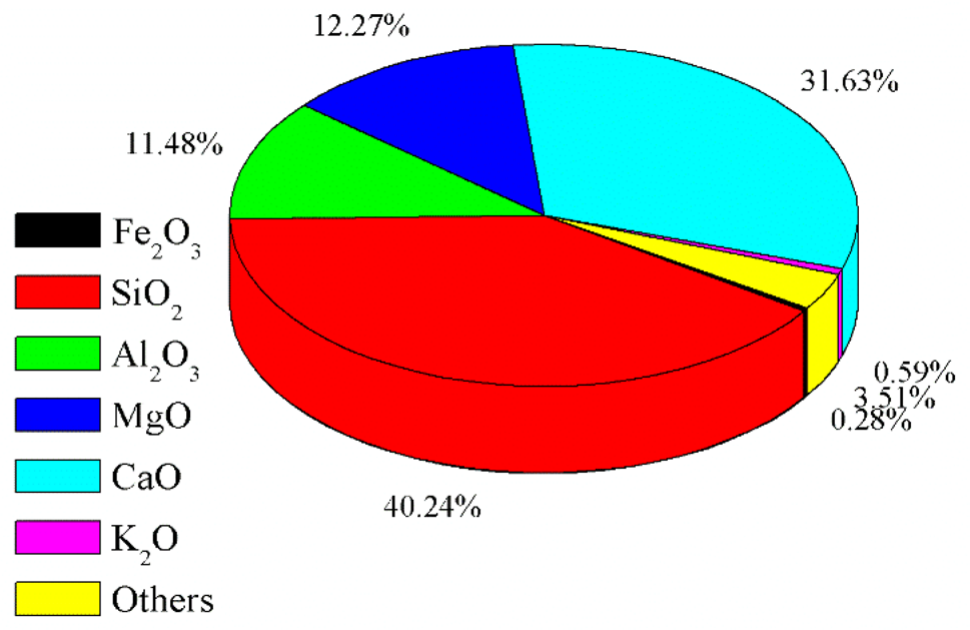
Chemical composition of steel slag particle electrodes.

Figure 3 displays percentages of chemical composition contained in the particle electrodes, which were observed by XRF. As seen, the steel slag was mainly composited of several kinds of oxides, including metallic oxides and transition-metal oxides. It was observed that the contents of silicon dioxide (SiO_2_) and calcium oxide (CaO) were relatively high, accounting for 40.24% and 31.63% respectively. Besides, the steel slag was also composed of 12.27% magnesium oxide (MgO), 11.48% aluminium oxide (Al_2_O_3_), 0.59% potassium oxide (K_2_O), 0.28% ferric oxide (Fe_2_O_3_) and others, indicating the complexity. Moreover, these existing metallic oxides and transition metal oxides participated in the reaction through heterogeneous catalysis, facilitating the adsorption and electroconduction of particle electrodes, and therefore enhancing the effects of electrolysis for decomposing pollutants^28^.

**Figure 4:**
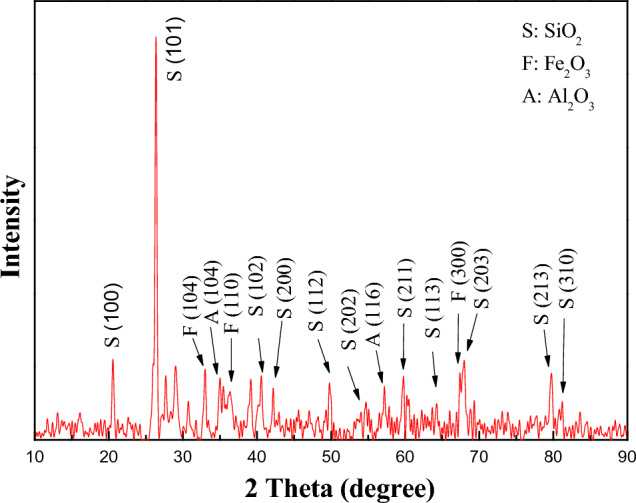
XRD pattern of steel slag particle electrodes.

The XRD pattern of steel slag particle electrodes were investigated and results are shown in Fig. 4. As could be seen, several pointed XRD peaks at 20.7°, 26.3°, 39.2°, 42.2°, 49.8°, 54.7°, 59.8°, 63.7°, 68.0°, 79.7° and 81.2° were detected, which were well indexed to (100), (101), (102), (200), (112), (202), (211), (113), (203), (213) and (310) planes for SiO_2_ with the Quartz crystal phase (JCPDS, 46–1045)^29^, respectively, suggesting SiO_2_ (Quartz) accounted for a large percentage of steel slag, which was in accord with XRF results. Besides, Fe_2_O_3_ with crystal phase of Hematite (JCPDS, 33–0664) could be detected in the particle electrodes, at 2 theta values of 33.0°, 35.5° and 64.3°, corresponding to (104), (110) and (300) planes^30^. It should be noted that the steel slags exhibited sight diffraction peaks with 35.0° and 57.2°, both indexed to (104) and (116) standard diffraction data for Al_2_O_3_ (JCPDS, 46–1212)^31^. In addition, some components of steel slag would not be detected in the XRD measurement, as its amorphous phases, or the complex composition of steel slag^32^. These steel slag particle electrodes showed sharp and intense diffraction peaks of various crystal phases, which could be beneficial for electrocatalytic removal of pollutants in the three-dimensional electrochemical oxidation system.

### Comparison of 3D and 2D electrochemical oxidation systems

**Figure 5:**
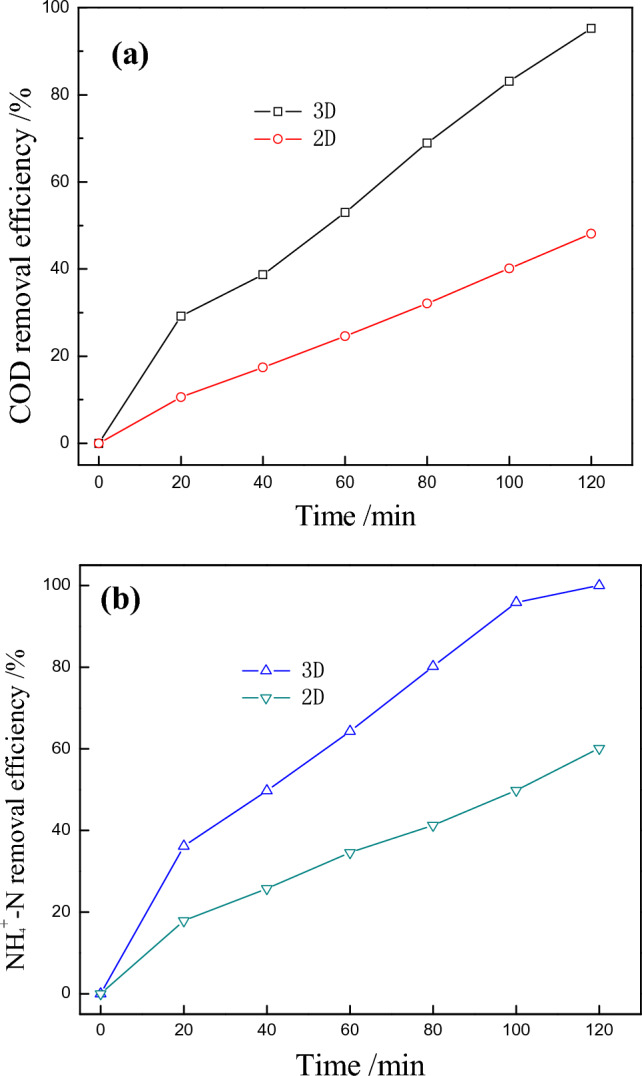
Comparison of COD and NH_4_^+^–N removal efficiencies in 2D and 3D electrochemical oxidation system. (interelectrode distance of 1 cm, current density of 20 mA·cm^−2^, initial pH value of 4.4, steel slags concentration of 0.30 g·mL^−1^).

Figure 5 shows COD and NH_4_^+^-N removal efficiencies for landfill leachate in the 3D and conventional 2D systems under same operating conditions, where the interelectrode distance of 1 cm, current density of 20 mA·cm^−2^ and initial pH of 4.4 were set. As displayed in Fig. 5a, b, high concentration and toxic landfill leachate could be degraded within 120 min in the 3D electrochemical oxidation system, with COD and NH_4_^+^-N removal efficiencies of 95.23% and 98.82% achieved. However, only 48.12% of COD and 60.12% of NH_4_^+^-N could be removed when decomposing landfill leachate in 2D system within 120 min, indicating that the steel slag particle electrodes greatly improved the degradations of landfill leachate, which might be attributed to the following aspects: (a) The steel slags had high specific surface area to be adhered by pollutants, which expanded the total reaction areas through which electrochemical reactions might take place. (b) By adding particle electrodes and applying the appropriate electric field, the particles formed many micro-electrodes with different charges on both ends, due to electrostatic induction, and therefore produced more hydroxyl radicals for further decomposing organics^33–35^.

### Optimization of parameters for three-dimensional electrochemical oxidation system

#### Optimization of interelectrode distance

**Figure 6:**
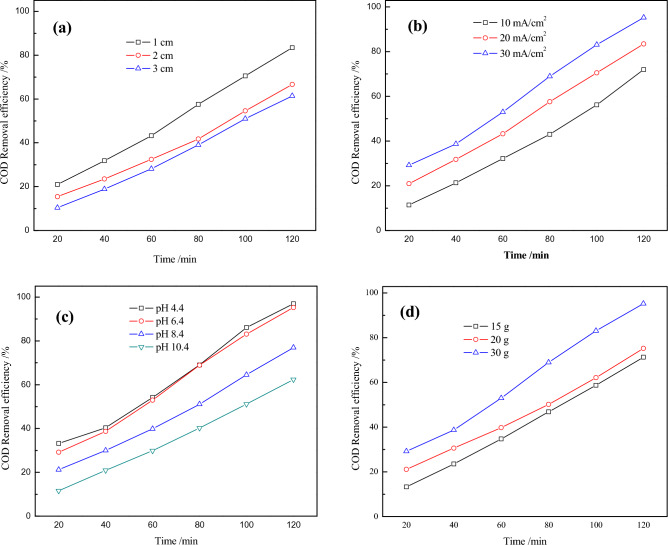
Effects of interelectrode distances (**a**, current density of 20 mA·cm^−2^, initial pH of 4.4, steel slags concentration of 0.30 g·mL^−1^), current densities (**b**, interelectrode distance of 1 cm, initial pH of 4.4, steel slags concentration of 0.30 g·mL^−1^), initial pH values (**c**, interelectrode distance of 1 cm, current density of 20 mA·cm^−2^, steel slags concentration of 0.30 g·mL^−1^) and steel slag.

The process in the electrochemical reactor was generally controlled by either electron/charge or mass transfer, which in turn was affected by the distance between the main electrodes. Specifically, for the 3D electrochemical oxidation system, the interelectrode distance also determined the repolarization extent of the particle electrodes^18^, which therefore was investigated for degradation test. Figure 6a displays the effects of three interelectrode distances (1 cm, 2 cm and 3 cm) on the COD removal efficiencies for landfill leachate under same operating conditions. Clearly, a reduction in interelectrode distance increased the COD removal efficiency. 83.41% of COD was removed at an interelectrode distance of 1 cm after 120 min degradation process, which was about 1.29 times larger than the COD degradation rate at 3 cm distance (61.41%), suggesting a much better performance was obtained at shorter interelectrode distances. As a result, the intensity of electric field as well as electron transfer rate increased at short interelectrode distances, while the distance for substance diffusion was reduced in the process^23^. Thus, the mass transfer would be facilitated and degradation efficiency might be enhanced as well^36^. Additionally, the longer distances between electrodes and tortuous path for ionic conductivity were expected to increase the potential of cell and on the surface of electrodes. The resistance of electrolyte path was reduced with decreased interelectrode distance, which decreased the ohmic loss to the electrolytic cell potential. Meanwhile, the potential drop along the steel slag was reduced. Besides, water oxidation was expected to become kinetically more facile than oxidation of dissolved species at more oxidative potential in the anode. Owing to these aspects, there were lower removal efficiencies for 2 cm and 3 cm experiments. Furthermore, 1 cm was selected as the optimal interelectrode distance.

#### Optimization of current density

The current density (j) was a primary parameter in contaminant removal and capital cost during electrochemical wastewater treatment^37^. Various current densities (10 mA·cm^−2^, 20 mA·cm^−2^ and 30 mA·cm^−2^) were applied for operating the optimized conditions. As seen from Fig. 6b, the COD removal efficiencies for landfill leachate increased along with j values. This was because larger amounts of hydroxyl radicals were generated and participated in the degradation process at higher current density. Although higher COD degradation rate of 95.23% could be achieved when the current density was 30 mA·cm^−2^, there would be some drawbacks: On one hand, side reactions, such as oxygen and hydrogen evolution, which competed with main reactions would occur at high j values, thereby leading to the deactivation of electrodes and shortening of service time^38^. On the other hand, higher j values could result in much more energy consumption, and increased operating cost^39^. Specifically, the maximum removal rate for COD in 120 min was 83.41% at the current density of 20 mA·cm^−2^, which was relatively high to be obtained. Accordingly, 20 mA·cm^−2^ was selected as the optimal current density for further experiments.

#### Optimization of initial pH value

The influence of initial pH value on degradation performance of landfill leachate in the 3D electrochemical oxidation system was then investigated. Figure 6c shows the COD removal efficiencies of four different initial pH values (4.4, 6.4, 8.4 and 10.4). It was observed that better removal efficiencies were obtained under mild acidic conditions (pH of 4.4 and 6.4), with over 95% of COD removed respectively. To explain these results, the acidic condition could decrease oxygen evolution from anodic oxidation, which was beneficial to electrode reactions. However, side reactions always occurred around the electrodes in the alkaline solution, causing inactivation of electrodes and therefore hindering the degradation reactions^19^. In addition, the steel slags which contained iron oxides would facilitate the decomposition of the landfill leachate, due to Fenton-like reaction that would occur at lower pH values^40^. Moreover, in order to maintain the acidic condition, the optimized initial pH value was set at 4.4.

#### Optimization of steel slag concentration

Steel slag particles with formation of charged micro-electrodes could result in high degradation efficiency in the 3D electrochemical oxidation system, which played an important role there. According to previous studies^23^, the characteristic excellent adsorption capacity for steel slags allowed the pollutants in the wastewater to concentrate on its surface, so the pollutants were easily and directly oxidized. Thus, the performance of degradation system was evaluated under various steel slag concentration (0.15 g·mL^−1^, 0.20 g·mL^−1^ and 0.30 g·mL^−1^) and other conditions were fixed (Fig. 6d). As expected, the COD removal rate for the landfill leachate increased from 71.29 to 95.23% within 120 min, along with increased steel slag dosage from 0.15 to 0.30 g·mL^−1^. This was because the amount of dissolved metal ions and solid–liquid interfaces in the reaction system increased as well. Noticeably, compared with other conditions, the performance was obviously enhanced when 0.30 g·mL^−1^ of steel slags was filled in the reactor, which might be explained by the sharp increase of saturation adsorption sites. Finally, the best set operating conditions were: interelectrode distance of 1 cm, current density of 20 mA·cm^−2^, initial pH value of 4.4 and steel slag concentration of 0.30 g·mL^−1^.

#### Possible removal mechanism

**Figure 7:**
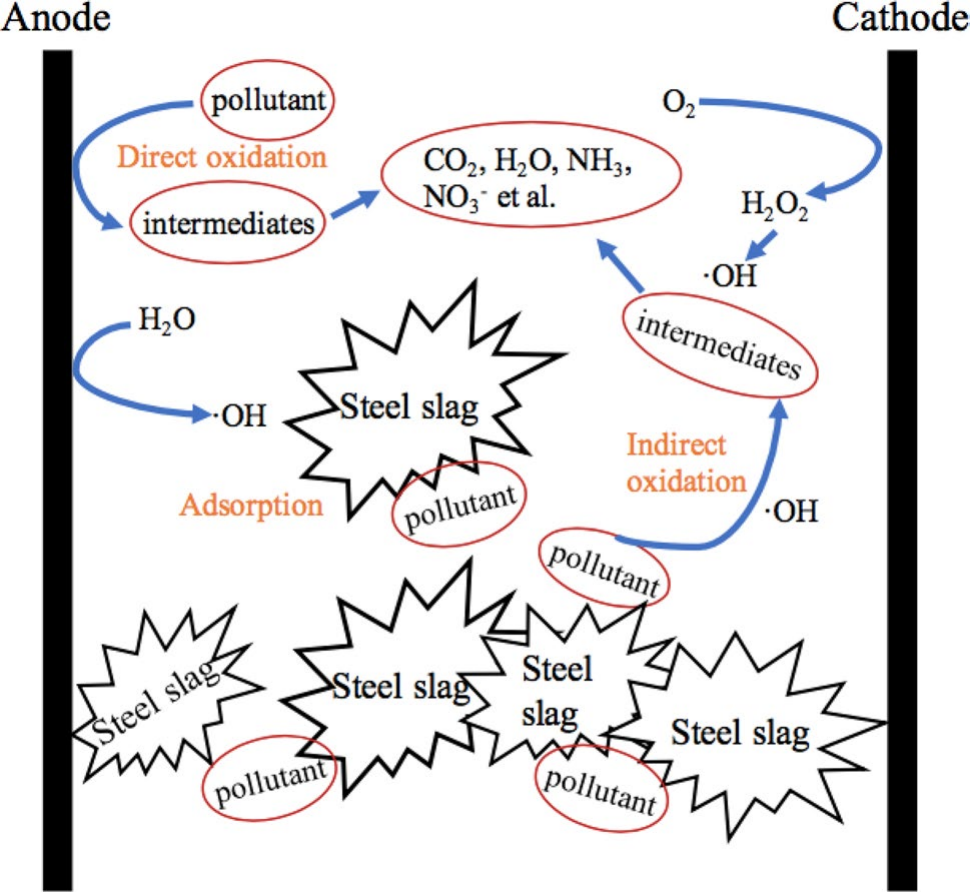
Possible degradation mechanism for pollutants in the three-dimensional electrochemical oxidation system.

The mechanism for pollutants removal generally included direct oxidation (direct electron transfer on the anode) and indirect oxidation (electro-generated oxidizing species) in the 3D electrochemical oxidation system^41,42^. In the system, the anode with electron transfer worked a lot for oxidizing pollutants^43^. In detail, both physisorbed and chemisorbed hydroxyl radicals were generated from the discharge of water, which subsequently participated in the oxidation process^44^. Besides, the introduction of steel slag to form a 3D system could expand the total reaction area, which facilitated the degradation. Moreover, and it could also accelerate the electron transfer efficiency, greatly improving the LFL decomposition. Considering these, the diagram for possible degradation mechanism of the landfill leachate is shown in Fig. 7 and the occurring reactions are listed as below^45,46^.

At the anode:

1 $${\text{H}}_{{2}} {\text{O}} \to \cdot {\text{OH }} + {\text{ H}}^{ + } + {\text{ e}}^{ - }$$

2 $${\text{2 H}}_{{2}} {\text{O }} - {\text{ 4 e}}^{ - } \to {\text{4 H}}^{ + } + {\text{ O}}_{{2}}$$

At the cathode:

3 $${\text{O}}_{{2}} + {\text{ H}}^{ + } + {\text{ 2 e}}^{ - } \to {\text{H}}_{{2}} {\text{O}}_{{2}}$$

4 $${\text{H}}_{{2}} {\text{O}}_{{2}} \to {2} \cdot {\text{OH}}$$

5 $${\text{2 H}}_{{2}} {\text{O }} + {\text{ 2 e}} \to {\text{H}}_{{2}} \uparrow \, + {\text{ 2 OH}}^{ - }$$

## Conclusions

In summary, the 3D electrochemical oxidation system with steel slag particle electrodes was constructed in this study for landfill leachate degradation. The process conditions were also optimized. For investigating the physicochemical properties of steel slag, XRF, SEM and XRD measurements were carried out and results indicated that steel slag was rough block, mainly consisting of SiO_2_ (Quartz crystal phase), which was beneficial for adsorption and electric conduction. Besides, when compared with 2D system, 3D electrolysis showed much better performance, with COD and NH_4_^+^-N removal efficiencies of 95.23% and 98.82% achieved in 120 min degradation. In addition, interelectrode distance of 1 cm, current density of 20 mA·cm^−2^, initial pH of 4.4 and steel slag concentration of 0.30 g·mL^−1^ were identified as optimal operating parameters. Finally, the mechanism for pollutants removal was also proposed in this study. The steel slag increased the reaction area and improved the electron efficiency as electrode particles, which made great contribution in the oxidation of LFL. The slag was thus environmentally friendly when used to develop three-dimensional electrochemical oxidation system for contaminant removal. The steel slag is thus an attractive option for particle electrode application.

## Data Availability

Data is available under reasonable request to the corresponding author.
